# Reply to Prof. Bigelow by Prof. Gunn

**Published:** 1870-02

**Authors:** Moses Gunn


### REPLY TO PROF. BIGELOW BY PROF. GUNN.

Editor Chicago Medical Examiner:—Allow me to reply to Prof. Bigelow's letter in the last number of your journal, in which he trusts that I will "acquit him of intentional injustice." I sincerely wish that the character of his letter was such as to make it easy for me to do so. He opens with the following sentence:—

"In a pamphlet which Prof. Gunn has rather widely distributed in this vicinity, and which has been brought to my notice since I recently returned from abroad," etc.

I trust that Prof. Bigelow did not think that I had omitted him in my distribution; for, I assure him, that I was more anxious to have him see my pamphlet, than any other one man; and I consequently mailed the first copy, sent out, to him.

As to the matter in controversy, it is a very simple question: did I anticipate Prof. Bigelow several years in recognizing the influence of the capsular ligament, including the ilio-femoral ligament, upon the *dorsal* and *ischiatic* varieties of luxation? This is distinctly what I claim: in proof of which I quote from a short article which I published in the *Peninsular Journal*, in 1853. Speaking of dorsal dislocation, I used the following language:—

"During the dislodgement of the bone, the superior and posterior portion of the capsular ligament is ruptured, through which the head protrudes; while, from the position of the limb at the instant of protrusion, *the anterior and inferior portion* is very much relaxed, thus allowing the head to rise easily over the rim of acetabulum. As soon as the head settles into its position upon the dorsum of the ilium, the direction of the limb is changed, and the untorn portion of the ligament becomes more tense, and, for this reason, the head of the bone can not be readily returned to its place, till the limb is again placed in a position to relax it."

In the same article, also, I used the following language:—

"This principle can be successfully applied to the reduction of the backward luxation of the femur into the ischiatic notch."

In 1856, I wrote as follows:—

"The upper and posterior half of the capsule was then cut away, leaving the *anterior and inferior half whole*, and the round ligament was divided. In this state, it will be seen that *all tissues* are entirely out of the way (and could neither afford protection against dislocation, nor impediment to reduction), except the *anterior and inferior half* of the capsular ligament."

I think my position, taken in the above quotations, covers, so far as *dorsal* and *ischiatic* luxations are concerned, the entire ground assumed by Prof. Bigelow, in reference to the *same dislocations;* notwithstanding, he says that I "show no knowledge of the existence of an ilio-femoral ligament." (Courteous, certainly!) The *constituent* character of the ilio-femoral ligament, in relation to the capsular ligament is proven, not only by the anatomy of the parts, and by reference to all anatomical descriptions of the same, but also by Prof. Bigelow himself, who says:—

"The anterior part of the capsule of the hip-joint is a triangular ligament, of great strength."

I beg the reader to notice the force of the language "part of;" and I appeal to any surgeon, whether in speaking of the capsular ligament of the hip-joint, he has mentally excluded the ilio-femoral fibres.

Referring to my claim, Prof. Bigelow says:—

"Prof. Gunn should remember, that although the greater may, in one sense, include the less, as a block of marble does a statue, yet there is no sentence or word in his whole pamphlet which alludes to the existence of an ilio-femoral ligament in the capsule."

Now, let us take, for an example, the dorsal luxation: does Prof. Bigelow claim that the capsule is completely torn asunder, with the exception of the ilio-femoral fibres? If so, why "circumduct," so as "to rupture in this way the whole of the rest of the capsule?" If *not* so, I would suggest that Prof. Bigelow's pet statuette is not yet chiseled, and that truth is better represented in the block as I left it.

I make no claim to Prof. Bigelow's ideas, in reference to other dislocations. As to them, we differ widely. How soon, or to what extent, I may hereafter adopt them, time, observation and thought will determine. But if, in the two specified, we are so far asunder in our views of the *lesion*, and its influence, why does Prof. Bigelow take pains to state that he "first met" my "pamphlet" several years after my (his) own theory was made public?" I would suggest that this *naive* remark is probably significant of Prof. Bigelow's own consciousness.

In addition to the above, permit me to append the following —I hope impartial and *strictly* fair—paragraphs, which I had already prepared as a postscript to my little pamphlet:—

"The part played by the *anterior* and *inferior* portion of the capsule of the hip-joint, in its untorn condition, in *dorsal* and *ischiatic* luxations of the head of the femur, both in producing the characteristic phenomena of these lesions, and in opposing ordinarily directed efforts to reduce them, was worked out by process of thought *and experiment.* But the idea, that in downward and forward luxations, the *upward* and *backward* half of the capsule remained untorn, and that, in forward luxations, the *posterior* half was unlacerated, was reasoned out, both from analogy and the manner in which these accidents occur. But no experiment was instituted to prove the position taken. I was, therefore, unprepared, in issuing this edition, to either take issue, or coincide with Prof. Bigelow's assertion, that the ilio-femoral portion of the capsule remained intact, in all the four standard dislocations of the hip-joint.

"I have, however, since then, in preparing to illustrate my lectures on this subject, during the current session, resorted to the following experiments, to-wit:—

"A fresh, middle-aged subject was selected, upon which I first produced the forward luxation. The characteristic signs were present. I dissected away the integument and superficial fascia. The fascia lata was found lacerated from its attachment to Poupart's ligament, and through the fissure was seen the shining surface of the femoral head; clearing away this fascia, the sartorius was found overlying the trochanter major; the psoas magnus and iliacus hugged closely the outer extremity of the neck. The head lay upon the origin of the pectineus, the outer edge of which was lacerated. Dividing and reflecting these muscles, the anterior surface of the neck and head of the femur are entirely exposed. The capsule *was completely torn from the whole anterior intertrochanteric line*, including the reinforcing fibres of the ilio-femoral, and split to its acetabular attachment.

"Upon the other side, I effected a downward luxation upon the obturator foramen. An examination revealed extensive rupture of the capsule, upon the side adjacent to the foramen, and an *intact condition of the ilio-femoral* and of the capsule opposite to the rupture. Careful inspection of the mechanism of the untorn portion of the capsule, including the reinforcing fibres of the ilio-femoral, revealed the fact, that the attachment of the upper end of the ilio-femoral being so far *above* the socket made its influence identical with that of the upper and untorn portion of the capsule proper. In *mechanical effect*, the ilio-femoral may be regarded, in this accident, as a constituent part of the *upper* portion of the ligament. Experiment, also, confirmed the correctness of my directions as to the method of reduction."

I would also remark, upon Prof. Bigelow's mode of reduction, that he seems to regard the ilio-femoral as the only thing of importance or use about the joint, and proceeds to "circumduct" and tear the rest of the capsule "with impunity." Perhaps the injuries already sustained by the joint may be generally added to "with impunity;" but would it not be better and more surgeon-like to avoid further laceration? I confess that I can see but little to choose, between the application of power, by means of the pulley, or by means of the lever (which latter is essentially Prof. Bigelow's mode), only, that by the latter plan, greater speed and efficiency are attained, by a more advantageous application of the power used. Of the two, the latter is capable of the greater mischief, and both are too barbarous to be resorted to, in most cases of recent luxation.

MOSES GUNN.

